# CACTUS: a computational framework for generating realistic white matter microstructure substrates

**DOI:** 10.3389/fninf.2023.1208073

**Published:** 2023-08-01

**Authors:** Juan Luis Villarreal-Haro, Remy Gardier, Erick J. Canales-Rodríguez, Elda Fischi-Gomez, Gabriel Girard, Jean-Philippe Thiran, Jonathan Rafael-Patiño

**Affiliations:** ^1^Signal Processing Laboratory (LTS5), École Polytechnique Frale de Lausanne (EPFL), Lausanne, Switzerland; ^2^CIBM Center for Biomedical Imaging, Lausanne, Switzerland; ^3^Radiology Department, Centre Hospitalier Universitaire Vaudois, University of Lausanne, Lausanne, Switzerland; ^4^Department of Computer Science, University of Sherbrooke, Sherbrooke, QC, Canada

**Keywords:** microstructure imaging, diffusion MRI, brain imaging, white matter, Monte-Carlo simulations, numerical phantom, synthetic substrates, high packing density

## Abstract

Monte-Carlo diffusion simulations are a powerful tool for validating tissue microstructure models by generating synthetic diffusion-weighted magnetic resonance images (DW-MRI) in controlled environments. This is fundamental for understanding the link between micrometre-scale tissue properties and DW-MRI signals measured at the millimetre-scale, optimizing acquisition protocols to target microstructure properties of interest, and exploring the robustness and accuracy of estimation methods. However, accurate simulations require substrates that reflect the main microstructural features of the studied tissue. To address this challenge, we introduce a novel computational workflow, CACTUS (Computational Axonal Configurator for Tailored and Ultradense Substrates), for generating synthetic white matter substrates. Our approach allows constructing substrates with higher packing density than existing methods, up to 95% intra-axonal volume fraction, and larger voxel sizes of up to 500μm^3^ with rich fibre complexity. CACTUS generates bundles with angular dispersion, bundle crossings, and variations along the fibres of their inner and outer radii and g-ratio. We achieve this by introducing a novel global cost function and a fibre radial growth approach that allows substrates to match predefined targeted characteristics and mirror those reported in histological studies. CACTUS improves the development of complex synthetic substrates, paving the way for future applications in microstructure imaging.

## 1. Introduction

Diffusion-weighted magnetic resonance imaging (DW-MRI) is a non-invasive technique used to study the microscopic structure of biological tissues *in vivo*. It is sensitive to the ensemble of water molecules (wherein each molecule follows a random motion pattern) as they interact with cellular surfaces (Simpson and Carr, 1958; Stejskal and Tanner, 1965; Bihan, 1995). This technique provides a valuable tool to study brain microstructure and its alterations following injury (Parizel et al., 2005; To et al., 2022) and neurological disease (van Gelderen et al., 1994; Budde and Frank, 2010; Narvaez-Delgado et al., 2019).

White matter is a crucial component of the brain, composed of highly organized axon bundles that interconnect cortical regions and subcortical regions (Brückner et al., 1996; Sporns, 2011). Various imaging techniques have been considered to characterise the white matter tissue microstructure in different species. For example, axon diameters have been measured in some white matter regions of the macaque monkey brain using histology and DW-MRI (Caminiti et al., 2013), and optical microscopy (Innocenti and Caminiti, 2017). These studies show that the estimated distribution of axon diameters is long-tailed, with a mean of around one micrometre. Recent studies that used high-resolution three-dimensional (3D) synchrotron X-ray nano-holotomography (Andersson et al., 2020) and 3D electron microscopy (Lee et al., 2019) found that axons are non-cylindrical and exhibit environment-dependent variations in diameter and trajectory. Alongside axon diameters, another relevant feature is the intracellular volume the axons occupy in a predetermined region. In histological postmortem data, the white matter intracellular space volume has been estimated as ranging between 60 and 85% of the brain volume for macaques (Stikov et al., 2015) and human adults (Syková and Nicholson, 2008). Interestingly, it goes as high as 70–95% in mice, as reported by light microscopy (Tønnesen et al., 2018), and cryo and chemical fixations (Korogod et al., 2015). It is speculated that this range might be influenced by the shrinkage of the extracellular space due to the fixation process (Dam, 1979; Bolduan et al., 2020).

Given the importance of studying white matter tissue microstructure *in vivo*, several DW-MRI models have been proposed (e.g., Murday and Cotts, 1968; Neuman, 1974; van Gelderen et al., 1994; Söderman and Jönsson, 1995; Stanisz et al., 1997; Assaf et al., 2004, 2008; Assaf and Basser, 2005; Alexander et al., 2010; Dyrby et al., 2011; Drobnjak et al., 2016; Jelescu and Budde, 2017; Kakkar et al., 2018; Novikov et al., 2018, 2019; Lee et al., 2020; Veraart et al., 2020, 2021; Harkins et al., 2021). However, validating these non-invasive techniques requires physical and numerical phantoms with a well-known microstructure (Campbell et al., 2005; Fieremans et al., 2008; Tournier et al., 2008; Fillard et al., 2011; Lavdas et al., 2013; Maier-Hein et al., 2017; Zhou et al., 2018; Schilling et al., 2019; Andersson et al., 2020; Lee et al., 2020; Rafael-Patino et al., 2020; Warner et al., 2023). Phantoms, in the context of this paper, are geometrical models of brain tissue structures that serve as a proxy or reference for evaluating the performance of imaging techniques. While physical phantoms have been widely used, they are often limited by their high costs and the impracticality of replicating axons' sizes and complex spatial arrangement. Therefore, numerical phantoms have emerged as the most popular validation technique for studying the complexities of diffusion phenomena in cases where analytical solutions are unavailable; because they only require a substrate that mimics the tissue of interest to simulate the displacements of water molecules and corresponding DW-MRI signal (Close et al., 2009; Côté et al., 2013; Neher et al., 2014). Nevertheless, the difficulty in Monte-Carlo simulations lies in accurately mimicking the geometry of white matter tissue (Hall and Alexander, 2009; Nilsson et al., 2012, 2017; Baxter and Frank, 2013; Plante and Cucinotta, 2013; Grussu et al., 2019; Truffet et al., 2020).

Various studies have proposed to generate numerical phantoms approaching the tissue's morphological complexity and density. For instance, two popular tools, MEDUSA (Ginsburger et al., 2019) and CONFIG (Callaghan et al., 2020), focus on generating specialized voxel-wise phantoms with microstructural geometries that replicate the properties of white matter. Recently, a tailored modification of Close et al. (2009) framework was used to build challenging substrates for the DiSCo challenge (Rafael-Patino et al., 2021), aimed to test fibre-tracking and connectivity methods on large-scale synthetic datasets from DW-MRI Monte-Carlo simulations. While these methods have provided valuable tools to characterise and simulate DW-MRI signals in numerical substrates, they still have important limitations regarding the maximum packing density and substrate size achieved. For instance, state-of-the-art frameworks can generate synthetic substrates with packing densities up to 75% (Ginsburger et al., 2019; Callaghan et al., 2020; Rafael-Patino et al., 2021), whereas the density found in histological data goes up to 95% in some regions (Korogod et al., 2015; Tønnesen et al., 2018). Moreover, they cannot sample substrate beyond 100μm^3^, which in turn restricts the sampling diversity achieved for morphological features (Romascano et al., 2018; Rafael-Patino et al., 2020). Therefore, the DW-MRI signals generated from these substrates may not accurately mimic the brain signals measured in white matter regions with higher packing densities.

To overcome these limitations, we introduce a novel computational workflow, CACTUS (Computational Axonal Configurator for Tailored and Ultradense Substrates), to generate synthetic fibres with rich microstructure characteristics. Expanding on previous methods (Close et al., 2009; Ginsburger et al., 2019; Rafael-Patino et al., 2020), we develop a novel numerical phantom generator for white matter substrates. CACTUS solves the high-density packing problem and achieves up to 95% intracellular volume fractions while efficiently generating substrate sizes up to 500μm^3^. Furthermore, CACTUS is highly customisable, capable of generating synthetic substrates with a wide range of characteristics, such as single-bundle (Stikov et al., 2015), bundle crossings (Tuch, 2004; Tournier et al., 2007; Schilling et al., 2017; Canales-Rodr-guez et al., 2019), orientation dispersion (Zhang et al., 2012; Daducci et al., 2015), gamma-distributed axon radii (Assaf et al., 2008; Sepehrband et al., 2016), non-constant longitudinal fibre-radii (Andersson et al., 2020), substrates with non-cylindrical fibres and tortuous surfaces (Lee et al., 2019), and myelin compartments (Mackay et al., 1994; Stikov et al., 2015; Canales-Rodr-guez et al., 2021). Through these features, CACTUS expands on the capabilities of existing substrate generation methods, providing a flexible and versatile tool for studying white matter microstructure in controlled environments.

## 2. Methods

**Figure 1:**
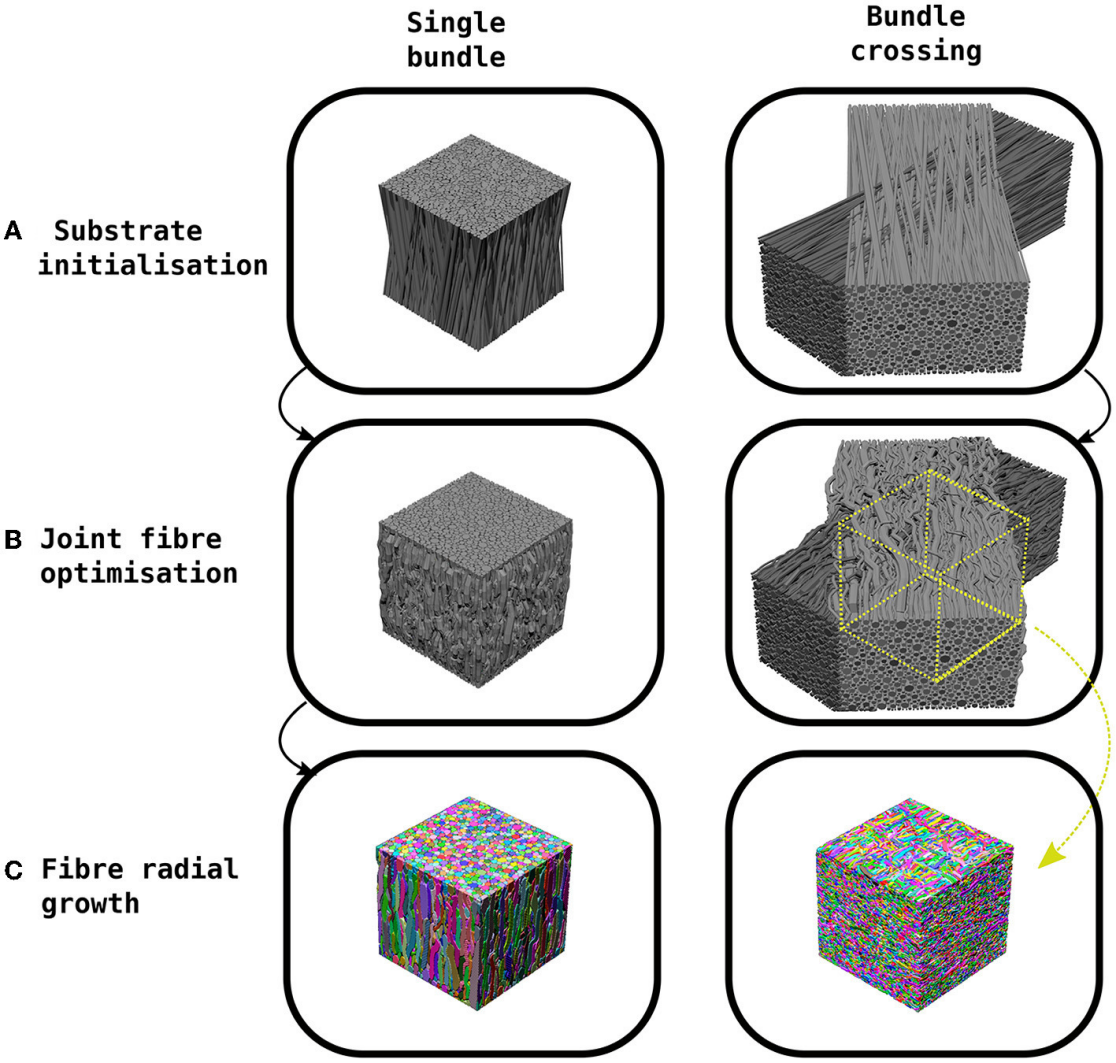
Example of the CACTUS method steps to create a synthetic substrate. **(A)** The substrate initialization orients fibres in a bundle to achieve a predefined mean angular dispersion (e.g., 15°). **(B)** The joint fibre optimization step removes fibre overlaps by adapting their trajectories and local radii. In the case of bundle crossings, the trajectories are trimmed to the centre of the crossing. **(C)** The fibre radial growth step further increases the fibre-packing density while keeping the predefined target radius distribution.

CACTUS generates synthetic substrates in three steps (see Figure 1): a) substrate initialisation, b) joint fibre optimisation, c) fibre radial growth (FRG).

Firstly, in the substrate initialization step, synthetic straight cylindrical fibres are initialized and parameterized inside a cuboid. In CACTUS, a single fibre population (bundle) is a group of fibres arranged cohesively along one main orientation. A bundle has two main properties: the average global dispersion, which is the mean angle between the main orientation of each fibre and the bundle, and the target radii distribution, from which the fibre radii are sampled.

In the second step, the joint fibre optimization, CACTUS extends previously proposed frameworks (Close et al., 2009; Ginsburger et al., 2019) based on local optimization. In our case, we aim to minimise a cost function that penalises some essential fibre properties such as overlapping, high curvature, increase in length, and promote compactness. Moreover, CACTUS introduces a new fibre parameterization based on capsules, which reduces the number of parameters needed to characterise fibre trajectories and handles fibre overlapping more efficiently. The resulting optimization problem is solved via a gradient descent algorithm (Duchi et al., 2011). During optimization, CACTUS prioritises removing fibre overlapping, while the penalization of curvature, length and promotion of compactness maintains a coherent fibre structure at all time-points.

Finally, the fibre trajectories are used to mesh the fibre surfaces in the fibre radial growth (FRG) step. The FRG also increases the packing density while keeping the correspondent fibre's parameterization structure using a discrete grid to seed, to grow, and to rearrange the fibre into the final substrates. The grid discretization defines the fibres' isosurface needed to compute the final surfaces with a marching cube algorithm (Lewiner et al., 2003).

### 2.1. Substrate initialization

Our substrate initialization algorithm enhances the circle two-dimensional (2D) packing algorithm proposed by Hall and Alexander (2009) to create a 3D packing of bundles. The algorithm creates a single bundle by initializing the fibres inside a cuboid of dimensions *L* × *L* × *H*. The endpoints of the fibres are contained within the *L* × *L* squared faces, while the orientation of the cuboid's height *H* and the bundle are aligned to the Z-axis. The algorithm packs 2D circles in the opposite faces of the cuboid, sampling radii from a gamma Γ(α, β) distribution (Assaf et al., 2008; Sepehrband et al., 2016), until the target density is met. At the same time, the algorithm packs the two opposite 2D circles to create an initialization such that the bundle reach the specified mean angular dispersion **η**. In scenarios where the target density exceeds 75%, an adjustment is made by shrinking the radii of the distribution. This allows the algorithm to continue packing until reaching a density of 75%. It is important to note that the radii will subsequently grow non-uniformly back to their original size during the execution of the algorithm while simultaneously achieving the desired final target density. In order to create a substrate with two bundles crossing at an inter-bundle angle of **θ**, two different bundles are initialized in their respective cuboids and subsequently rotated and translated are applied. Figure 1 shows examples of a single bundle and a bundle crossing initialization. Finally, we parameterise each fibre's skeleton as the trajectory of its centre of mass. This trajectory is defined by several control points connecting the two endpoints sampled during the packing algorithm, where each point has a corresponding radius.

### 2.2. Joint fibre optimization

Once the substrate is initiated as described in Section 2.1, fibres may overlap. CACTUS employs an optimization method to readjust the fibre trajectories and disentangle overlaps by defining several cost functions. These cost functions, inspired by Close et al. (2009) and Ginsburger et al. (2019), help to regularise and obtain coherent fibre structures with the specified target properties. Ordered by priority of penalization, these target properties are as follows: (i) fibre overlapping (see Section 2.2.1), (ii) high curvature, (iii) increased fibre length, (iv) changes in radii, and (v) compactness. The optimization algorithm alternative between two steps: first minimises the overlapping cost function, then the subsequent step aimed at minimizing the remaining cost functions. An algorithm requirement is to identify a solution that exhibits no overlaps. Once a solution without overlaps is achieved, the algorithm iterates further to reduce (when possible) the penalization associated with the remaining cost functions while maintaining the absence of overlaps.

In the following subsection, we introduce the novel parameterization and overlapping cost function based on capsules, which is a key contribution of our work. As the remaining cost functions are relatively straightforward and similar to those in previous studies, we have provided their definitions in Supplementary material (section joint fibre optimization).

#### 2.2.1. Fibre capsule-parameterization

**Figure 2:**
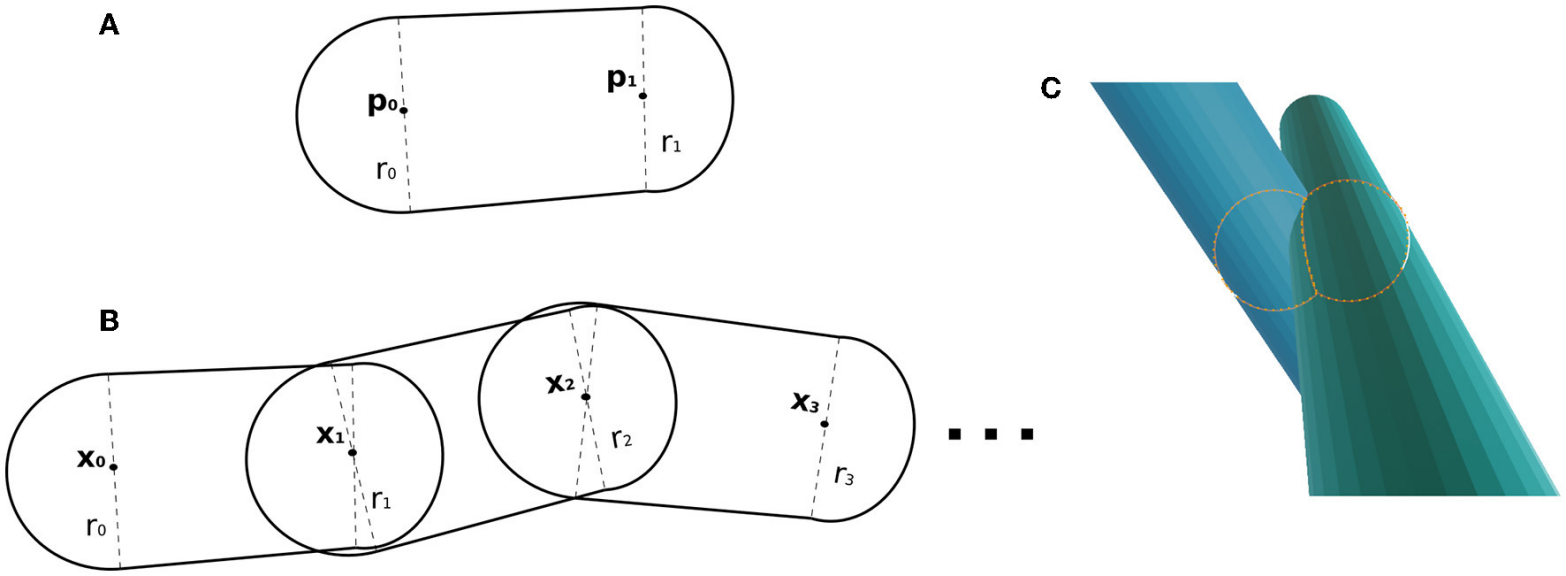
**(A)** Capsule example, whose parameters are two points and a radius at each control point. **(B)** Example of fibre as a chain of capsules. Two adjacent capsules share a control point (position and radius parameters). **(C)** Illustrative example of the key components within the overlapping cost function. It showcases the intersection of two capsules, with two spheres representing the intersection region between the capsules.

Fibres are parameterized as skeletons made of 3D control points. In the overlapping cost function, every pair of consecutive points in the skeleton forms a capsule, defined with the set of parameters [**p_0_**, **p_1_**, *r*_0_, *r*_1_], where **p_0_**, **p_1_** ∈ ℝ^3^ are the initial/ending points of the capsule, and *r*_0_, *r*_1_ ∈ ℝ are their respective radius (see Figure 2A). In our scenario, the length of a capsule (distance form *p*_*i*_ to *p*_*i*+1_) is not restricted, but we suggest the ranges between $\frac{1}{2}r_{i}$ up to 2*r*_*i*_, and the change sampling frequency increases as the radii decrease.

In this framework, a fibre parameterization can be defined as a chain of capsules (see Figure 2B). The fibre $S^{a}$, with *m*_*a*_ control points, is composed of the capsules determined by the subsequent point pairs as $\left[x_{i}^{a},x_{i+1}^{a},r_{i}^{a},r_{i+1}^{a}\right]$ with control points $\left\{x_{0}{}^{a},x_{1}{}^{a},\ldots x_{m_{a}-1}{}^{a}\right\}\subset {\mathbb{R}}^{3}$ and associated radius $\left\{r_{0}^{a},r_{1}^{a},\ldots r_{m_{a}-1}^{a}\right\}\subset \mathbb{R}.$

#### 2.2.2. Overlapping cost function

The overlapping cost function handles the fibre collision by identifying overlaps from two capsules from two different fibres. In CACTUS, the detection step of capsule intersection is a generalization of the cylinder-to-cylinder collision detection (Van Verth and Bishop, 2015). We define the overlapping cost function between two capsules by computing the overlapping of the closest spheres centred in the capsules, as Figure 2C shows. Formally, the closest points between two given capsules [***p*_0_, *p*_1_**, *r*_*p*_0__, *r*_*p*_1__], [***q*_0_, *q*_1_**, *r*_*q*_0__, *r*_*q*_1__], with ***p*_0_**, ***p*_1_**, ***q*_0_**, ***q*_1_** ∈ ℝ^3^ and , *r*_*pi*_, *r*_*qj*_ ∈ **R**, are the points centred in the capsule ((1 − *t*_*p*_)***p*_0_** + *t*_*p*_***p*_1_**) and, ((1 − *t*_*q*_)***q*_0_** + *t*_*q*_***q*_1_**), where *t*_*p*_, *t*_*q*_ are found by the following minimization problem:


(1) $$g(t_{a},t_{b};p_{0},p_{1},q_{0},q_{1})\;=\;∥(1-t_{a})p_{0}+t_{a}p_{1}-(1-t_{b})q_{0}-t_{b}q_{1}{∥}^{2}$$

(2) $$\begin{array}{l} t_{p},t_{q}:\;=\underset{t_{a},t_{b}}{\text{ arg min}}\;g(t_{a},t_{b};\;p_{0},p_{1},q_{0},q_{1}),\\ \;s.t.\;0\leq t_{a},t_{b}\leq 1 \end{array}$$

which has a closed-form solution.

After finding the values *t*_*p*_, *t*_*q*_ that define the closest centre points between two capsules of different fibres, their overlapping cost function is defined as:


(3) $$\begin{array}{l} f_{1}\left(p_{0},p_{1},q_{0},q_{1},r_{p_{0}},r_{p_{1}},r_{q_{0}},r_{q_{1}};t_{p},t_{q}\right)=\\ \left\{ \begin{array}{l} D^{2}∥p_{0}-p_{1}\text{​}∥∥q_{0}-q_{1}∥r_{p}r_{q},\text{ if }D\geq 0\\ 0,\text{ if }D<0 \end{array}\right. \end{array}$$

where,


(4) $$D:\;=\left(1-\frac{∥c_{1}-c_{2}∥}{r_{p}+r_{q}}\right),$$

(5) $$c_{1}:\;=(1-t_{p})p_{0}+t_{p}p_{1},$$

(6) $$c_{2}:\;=(1-t_{q})q_{0}+t_{q}q_{1},$$

(7) $$r_{p}\text{ : = }(1-t_{p})\;r_{p_{0}}\text{+ }t_{p}r_{p1},$$

(8) $$r_{q}\text{ : = }(1-t_{q})\;r_{q_{0}}\text{+ }t_{q}r_{q1},$$

and *t*_*p*_, *t*_*r*_ are the minimal values from the function in Equation 2.

Consequently, the total overlapping cost function in a substrate is computed by adding the evaluated cost of all possible pairwise capsule combinations. If capsules overlap, a penalization is added; otherwise, it is set to zero.

#### 2.2.3. Implementation details

At last, we mention the technical implementation details of the joint-fibre optimization algorithm, including strategies for reducing computational complexity and the use of specific data structures. Firstly, in the total overlapping cost function, the capsule-to-capsule comparison is a $O\left(n^{2}\right)$ problem. To improve computational time, we implemented a fixed-radius-cell data-structure (Turau, 1991) for nearest neighbours queries, reducing the problem to O(n). Since all the cost functions are analytical, we calculated their analytical derivatives for the gradient descent algorithm (see Supplementary material, section joint fibre optimization). We used the adaptative gradient Adagrad (Duchi et al., 2011), iterating until there were no overlapping fibres. All the cost functions, queries, and gradients calculations were implemented in C++ (Stroustrup, 1999) and parallelized with OpenMP (Chandra et al., 2001). To handle bundle crossings, we trim the optimized fibre trajectories to keep only a subregion with fibres that truly belong to the crossing, as shown in Figure 1. This step eliminates boundary fibres that may not fully represent the crossing characteristics.

### 2.3. Fibre radial growth

#### 2.3.1. FRG description

After completing the substrate initialization and joint fibre optimization steps, it follows to compute the fibre mesh. Previous studies have managed to achieve a fibre density up to 75% (Altendorf and Jeulin, 2011; Mingasson et al., 2017) with cylindrical-shaped fibres and gamma-distributed diameter, and up 75% with non-cylindrical shaped fibres (Callaghan et al., 2020). In this study, we propose a new method, called Fibre Radial Growth (FRG), to obtain higher packing density and complex axon morphologies beyond the cylindrical shape. The FRG algorithm discretises the 3D space that the fibres occupy to define individual masks for each fibre in it. The FRG algorithm begins to generate the fibre masks by randomly placing seed points within all capsule fibres. These seed points grow iteratively by adding neighbouring points to the fibre mask, employing a breadth-first-search approach through the grid. The seeds grow for a fixed number of iterations as long as they do not interfere with other fibres' boundaries. The propagation through random initializations avoids uniform growth and adds irregularities to the fibre shape, allowing tortuous surface reconstructions in the fibre surfaces. Since the seeding is done inside capsules, the final axon radius in the mesh is related to the radii used in the capsules. We employ two distinct seeding strategies to manage the *radius variation effect* in our study. The first one depends on the strategy of seeding in the FRG, which depends on how we seed points within the capsule and then grow the seeding points. We can achieve radii variations in intervals like [−*r*_*i*_/2, +*r*_*i*_/2] or (−∈, +∈) depending on whether we decide to seed more randomly or uniformly within the capsule.

In the second case, when we aim to increase the radii variation further in the (−*r*_*i*_/2, *r*_*i*_/2) range, we modify the fibre initialization step. We can define specific patterns in the radii of the fibres' capsules. For example, to incorporate a radii periodicity oscillation, we can set the radii at the start of the capsule to be 1μm and increase the end radii to 2μm. Then, in the subsequent capsule, we can choose to maintain the radii at 2μm or revert them to 1μm, based on the desired frequency of change specified by the user.

Once the FRG step is completed, the fibre density of the particular configuration inputted is maximized. We generate the fibre's outer surface mesh using the marching cubes algorithm (Lewiner et al., 2003; Pedregosa et al., 2011) applied to the fibre mask. This algorithm produces a mesh object consisting of vertices and triangles. Then, we applied a Laplacian smoothing (Herrmann, 1976; Sorkine et al., 2004; Sullivan and Kaszynski, 2019) to remove sharp angles, and finally decimate the mesh to reduce the number of triangles without affecting the morphology of the substrates (Shekhar et al., 1996). Subsequently, we generate a new mesh representing the fibre's inner surface by eroding the previously estimated outer grid and following the same procedure for the meshing. The space between these two surfaces defines the myelin volume.

#### 2.3.2. Implementation details

Finally, we would like to elaborate on the technical implementation details of the FRG algorithm to mention the specific design choices we made to ensure its computational efficiency. FRG is implemented in Python (Van Rossum and Drake, 2009), parallelized with its multiprocessing ibraries (McKerns et al., 2012), and compiled with Numba (Lam et al., 2015). Image 3D processing and meshing are done using van der Walt et al. (2014), Sullivan and Kaszynski (2019), and Hess (2010). Moreover, the FRG is designed to run a ball-tree structure (Moore et al., 2003) from the Sklearn library (Pedregosa et al., 2011) as a preprocessing to store fibres and their interactions. The fine-tuned FRG algorithm's design allows for the independent execution of fibre growth and meshing on multiple computers in a distributed manner, eliminating the need for multi-thread or computer synchronization.

## 3. Experiments

To evaluate the performance of CACTUS, we designed a comprehensive set of substrates with specific geometries. Each experiment below involves several metrics essential for quantifying the microstructure properties of the brain white matter. The metrics include the axon volume fractions, the radius distribution per substrate, the radii change along the fibres, the myelin volume, the g-ratio, the orientation dispersion and bundle crossings. Finally, we conducted testing on the generated substrates and performed Monte-Carlo diffusion simulations to assess their usability and explore the signal decay characteristics associated with these substrates.

### 3.1. Maximum fibre volume fraction

In our first experiment, we aim to explore the macro-structural parameters of substrates, such as substrate size (i.e., the voxel size in MRI experiments), fibre dispersion, two bundle crossings, and the ability to create high-density packing substrates. We assess the maximum fibre volume fraction that CACTUS achieves in two scenarios: a single bundle and two bundles. In the single bundle case, we generated six substrates with mean angle dispersions of 0, 5, 10, 15, 20, and 25°, respectively. In the two bundles case, we generated five crossing substrates with inter-bundle angles of 30, 45, 60, 75, and 90°, and the fibres of each bundle were initialized with a mean angle dispersion of 5° around the main bundle orientation.

### 3.2. Substrates targeting predefined microstructure features

The following two paragraphs describe experiments conducted to explore the ability of CACTUS to replicate desired microstructural parameters into its synthetic substrates. These parameters include the axon volume fraction (AVF), myelin volume fraction (MFV), g-ratio, and radii distribution. We compare the reference values taken from previous histological studies and those achieved by CACTUS.

In the second experiment, we created a series of synthetic substrates that emulate the histological values reported by Stikov et al. (2015) in various white matter regions. Specifically, the target characteristics are the fibre volume fraction, myelin volume fraction, and aggregated g-ratio, $g=\sqrt{1-MVF/FVF}$ (Stikov et al., 2015). In our scenario, the axon volume fraction (AVF) is the volume of the fibre inner surface. The myelin volume fraction (MVF) represents the volume of the space between the inner and outer fibre surfaces. The fibre volume fraction (FVF) is the sum of AVF and MVF.

In the third experiment, we investigated the effect of substrate size on radii distribution. To measure the radii distribution for each fibre, we cut the mesh skeleton in an orthogonal plane at regular 1μm intervals and calculated the cross-sectional area of the polygon defined by the plane. The equivalent fibre radius is defined as the radius of a circle with the same area as the polygon (Lee et al., 2019). The global radii distribution per substrate was computed using the mean radius for each fibre.

### 3.3. CACTUS substrates usage for Monte-Carlo simulations

The final experiment aims to evaluate the usability of CACTUS substrates in Monte-Carlo diffusion simulations. Synthetic DW-MRI data is generated using meshes obtained from previous experiments. This experiment aims to assess the feasibility and reliability of utilizing CACTUS meshes in Monte-Carlo simulations and examine the resulting DW-MRI signals of such substrates.

To simulate diffusion within non-permeable tissue, we utilized the MCDC Simulator (Rafael-Patino et al., 2020). In this context, the diffusion process within distinct biological structures was assumed to contribute independently to the DW-MRI signal. As a result, the intracellular and extracellular signals were generated separately and combined to generate the overall signal.

The four substrates simulated are composed of ~8,500 fibres. The fibres' outer diameter ranges from 0.5 to 4 um, sampled from a gamma distribution with parameters θ = 1.1, κ = 0.5. Each fibre's inner diameter is calculated from the following log-curve found in Lee et al. (2019).

The simulation substrates have a 300μm^3^ volume, split in an image size of (42 × 42 × 42) voxels of 7.14μm resolution. Within each voxel, the signal is simulating using random particle sampling with a density of one particle per cubic micrometre Rafael-Patino et al. (2020, 2021) and Romascano et al. (2018) showed that it is a sufficient number of particles to obtain a robust estimation of the diffusion signal in complex fibre geometries.

Particles initiated within the inner diameter of the fibres and outside the outer diameter of the fibres were used to generate the DW-MRI signal. The particles initiated between the outer and inner diameters were discarded because, in this case, we are not simulating the diffusion in the myelin compartment. This was done as previously in the DiSCo Challenge (Rafael-Patino et al., 2021), where no diffusion contrast is assumed in the myelin compartment, however T2 effects could be considered if necessary. The diffusion coefficient, which is user-defined, was fixed to $D=0.6\times 10^{-3}\frac{{\text{mm}}^{2}}{\text{s}}$ (corresponding to an *ex-vivo* diffusivity), for intra and extracellular compartments.

The DW-MRI protocol is based on the protocol of HCP (Fan et al., 2016). It contains four shells of 50 directions with *b*-values of (1,000, 2,000, 3,000, and 4,000) $\frac{\text{s}}{{\text{mm}}^{2}}$, and five directions (to calculate parallel and radial decay) with 20 *b*-values uniformly distributed from (500 to 10,000 ) $\frac{\text{s}}{{\text{mm}}^{2}}$. The protocol values are fixed for TE = 0.057 s, Δ = 21.8 ms, and δ = 12.9 ms.

## 4. Results

### 4.1. Maximum fibre volume fraction

**Figure 3:**
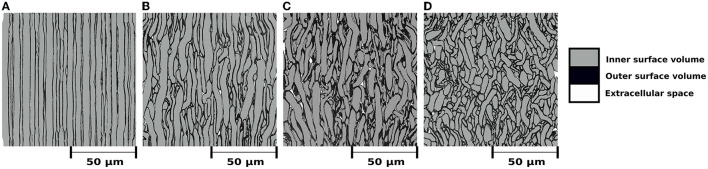
**(A–D)** Mesh's renders of cross-sections with dimensions of (100μm)^2^ to visualize the internal morphology of four substrates with a single bundle, each with different mean angular dispersion. For all cases, the outer surface volume is colored black. The inner surface volume is superimposed over the outer volume and colored gray. White represents the extracellular space, i.e., the volume not occupied by any fibre. All bundles are vertically aligned, and the substrates were built to have a mean angular dispersion of **(A)** 0°, **(b)** 5°, **(C)** 10°, and **(D)** 20°, respectively.

Figure 3 shows the internal morphology of four substrates consisting of a single bundle with a dispersion of 0, 5, 10, and 20°, respectively. All the substrates were generated with dimensions of 500μm^3^. Table 1 (top panel) reports the substrate characteristics, including the number of fibres, the obtained fibre volume fraction, and the dispersion parameters. We note that the maximum fibre volume fraction decreased from 94.7 to 90.8% as the dispersion increased from 0 to 25°.

**Table 1:** Substrate characteristics, including the number of bundles, mean dispersion angle, mean inter-bundle crossing angle, number of fibres per substrate, and fibre volume fraction (in per cent), respectively.

**Nbr of Bundles**	**Bundle dispersion (η)**	**Crossing angle (θ)**	**Nbr of fibres**	**FVF**
1	0°	-	31,954	94.7%
1	5°	-	31,023	93.4%
1	10°	-	30,241	92.6%
1	15°	-	30,412	92.2%
1	20°	-	31,161	91.8%
1	25°	-	31,863	90.8%
2	5°	30°	30,026	93.9%
2	5°	45°	30,712	93.3%
2	5°	60°	31,023	93.5%
2	5°	75°	31,152	92.3%
2	5°	90°	30,245	92.2%

The top and bottom panels correspond to the substrates with a single bundle and two bundles. Each row represents a different substrate.

**Figure 4:**
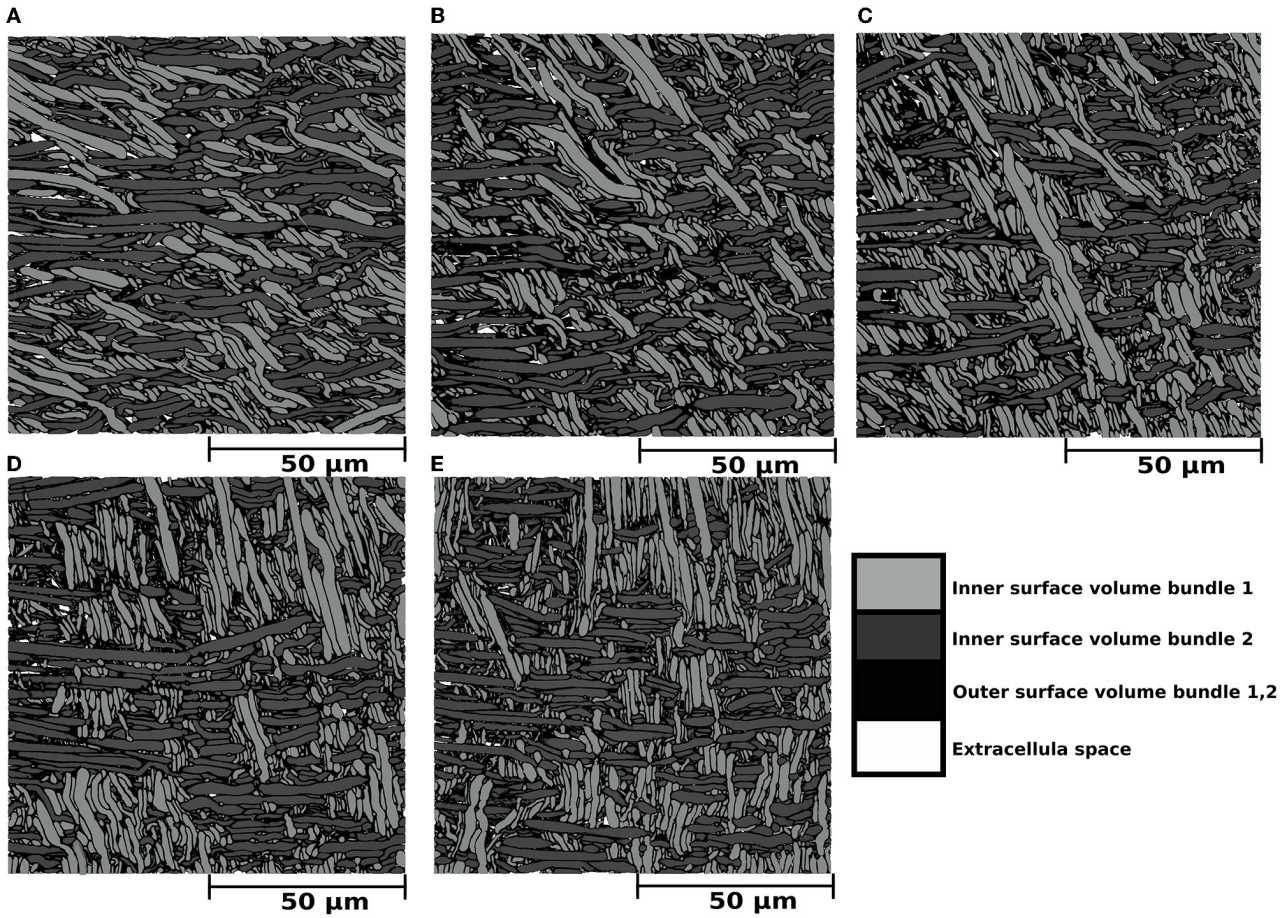
**(A–E)** Mesh renders of cross-sections with dimensions of (100μm)^2^ portraying the internal morphology of five substrates consisting of two bundles with different inter-bundle angles. In all cases, the outer surface volume of bundle 1 and bundle 2 is displayed in black. The inner surface volume is superimposed over the outer volume and colored light gray for bundle 1 and dark gray for bundle 2. The extracellular space, representing the volume not occupied by any fibre, is colored white. The dark gray fibres are aligned parallel to the *X* axis, and the light gray fibres crossed at angles of **(A)** 30°, **(B)** 45°, **(C)** 60°, **(D)** 75°, and **(E)** 90°. The outer volume, which is defined by the outer surfaces minus the inner volume, is colored in black for both bundles. Extra axonal space is colored in white.

Results from the experiment generating bundle crossings with different inter-bundle angles are depicted in Figure 4 and Table 1 (bottom panel). Figure 4 displays a cross-section of the substrates, where each bundle has a distinctive color for visualization purposes. Although local perturbations in fibre trajectories (on the order of 5°) may occur in the substrates due to the high fibre packing, the average bundle orientation is sustained. The bottom panel of Table 1 reports the fibre volume fraction of these bundle crossing substrates with inter-bundle angles of 30, 45, 60, 75, and 90°. For all the evaluated substrates, the fibre volume fraction remains nearly constant at ~93% (92.2 − 93.9%).

### 4.2. Substrates targeting predefined microstructure features

#### 4.2.1. Axon volume fraction, myelin volume fraction, and g-ratio

**Figure 5:**
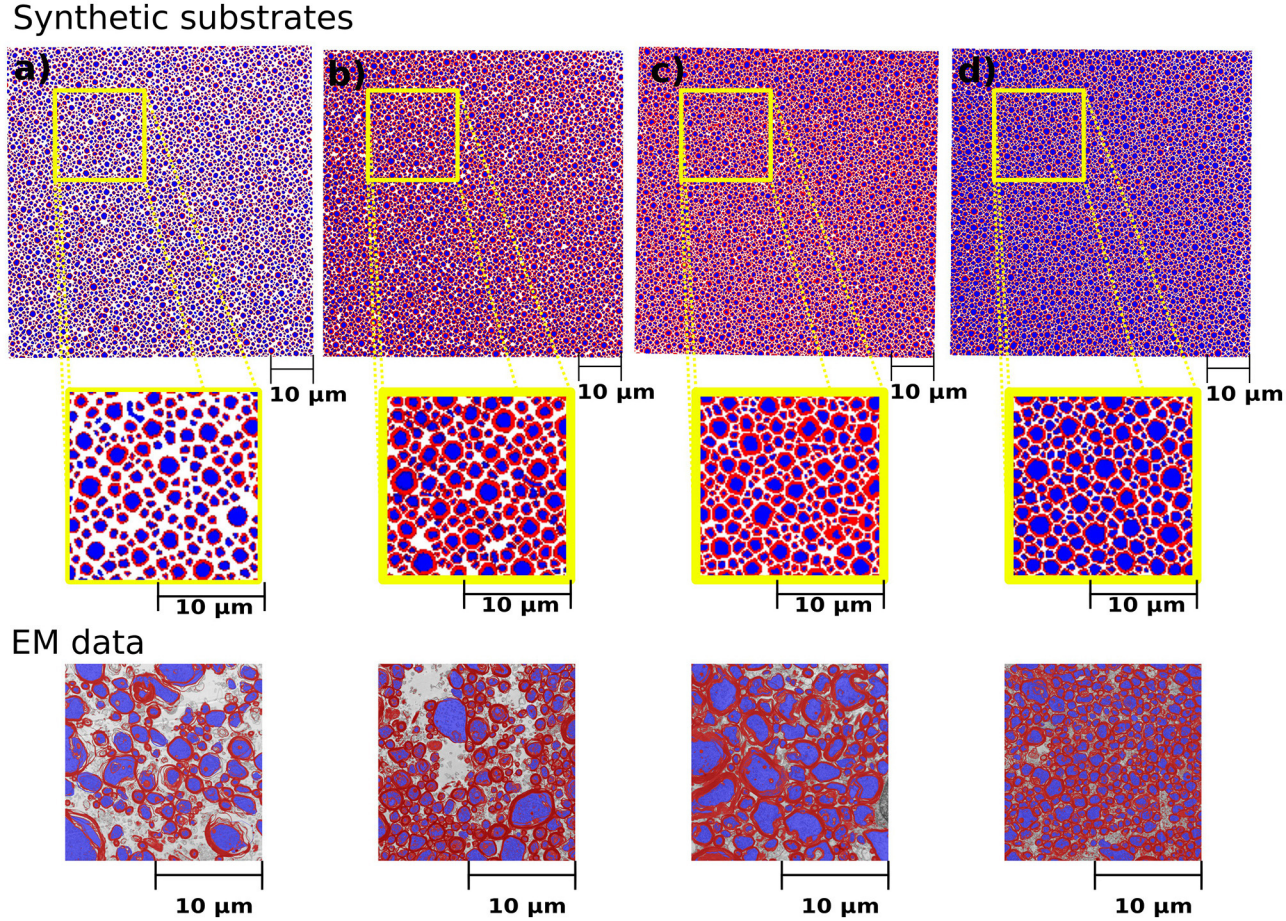
Cross-sections of the synthetic substrates constructed to match the statistics of the histological values reported in Table 2. The substrate dimension is 300μm^3^. For visualization purposes, the axonal space is colored blue, and the myelin is red, and extra axonal space is colored white. **(a–d)** Correspond to the same substrates shown in Table 2. The bottom panel shows the representative histological images courtesy of Prof. Nikola Stikov and Dr. Jennifer Campbell. The EM images are used to show the similarities in fibre shape and packing.

We simulated various substrates of a single bundle to mimic microstructure properties previously reported in Stikov et al. (2015). The histological values used as a reference are the myelin volume fraction (MVF), fibre volume fraction (FVF), axonal volume fraction (AVF=FVF-MVF), and g-ratio. The values achieved by CACTUS are shown in Table 2. The difference between the target and obtained substrate properties was lower than 2% in all cases. Examples of the generated substrates and histology data are shown in Figure 5. Electron microscopy images were generously provided by Prof. Nikola Stikov and Dr. Jennifer Campbell, and are used to highlight the geometric similarities of synthetic fibre shapes. On average, for these substrates of 300μm^3^, the meshes had around 46 million vertices and 92 million faces, and the file size is of 5.1 Gigabytes.

**Table 2:** Target microstructure histological properties (left) reported in Stikov et al. (2015), and corresponding properties of the substrates generated by CACTUS (right).

	**Target**				**Achieved**			
**Substrate**	**AVF**	**MVF**	**FVF**	**g-ratio**	**AVF**	**MVF**	**FVF**	**g-ratio**
**(a)**	**25**	**35**	**60**	**64.5**	**26.0**	**36.0**	**62**	**64.7**
(b)	25	43	68	60.6	26.3	43.6	69.9	61.3
(c)	31	44	75	64.2	32.2	43.8	76.07	65
(d)	39	37	76	71.6	41.2	35.0	76.0	73.5

The axon volume fraction (AVF) is the volume of the inner axon surface. The myelin volume fraction (MVF) represents the volume of the space between the inner and outer axon surfaces. The fibre volume fraction (FVF) is the sum of AVF and MVF. The aggregated g-ratio, $g=\sqrt{1-MVF/FVF}$ is equal to the mean inner and outer axon radius ratio for all the fibres in the substrate.

#### 4.2.2. Radii distribution and substrate size

**Figure 6:**
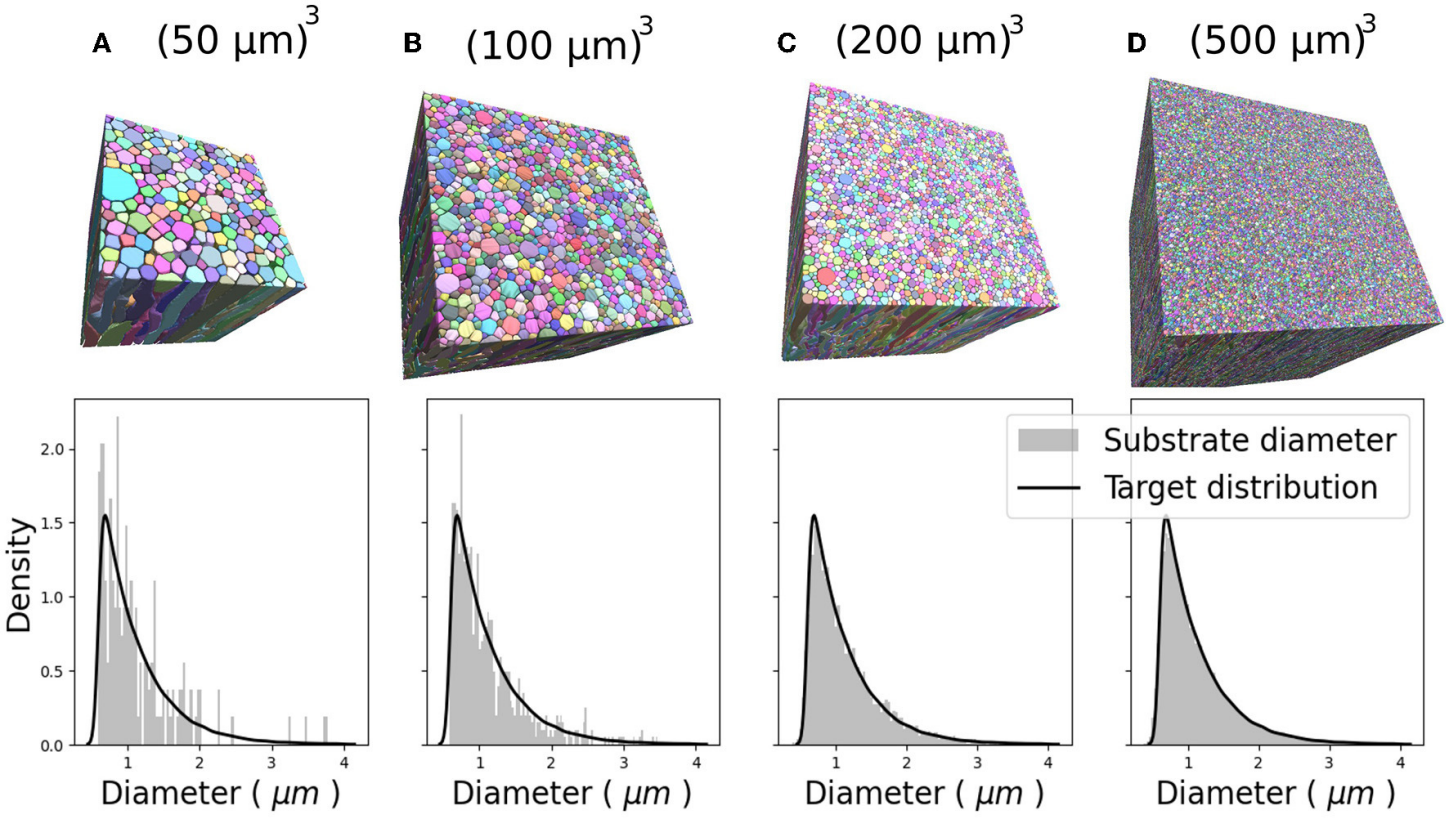
Four 3D substrates of varying sizes: **(A)** 50^3^, **(B)** 100^3^, **(C)** 200^3^, and **(D)** 500^3^ (μm)^3^, with 341, 1,316, 4,859, and 33,478 fibres, respectively. The empirical and target radii distributions are displayed on the bottom of each substrate. The empirical distribution better approximates the target distribution as substrate size increases.

Figure 6 show the CACTUS substrates with different sizes, ranging from 30 to 500μm^3^, and the target and empirical radius distributions obtained for each substrate. The empirical radius distributions closely replicated the targeted ones for substrates equal to or bigger than 200μm^3^. The optimization algorithm step ran for ~4 h for the largest substrate (right panel) on a node with 64 cores (2.4 GHz) and 400 Mb of RAM. The reconstruction time of the FRG algorithm was ~1 min per fibre, using one core with 500 Mbs of memory per core.

**Figure 7:**
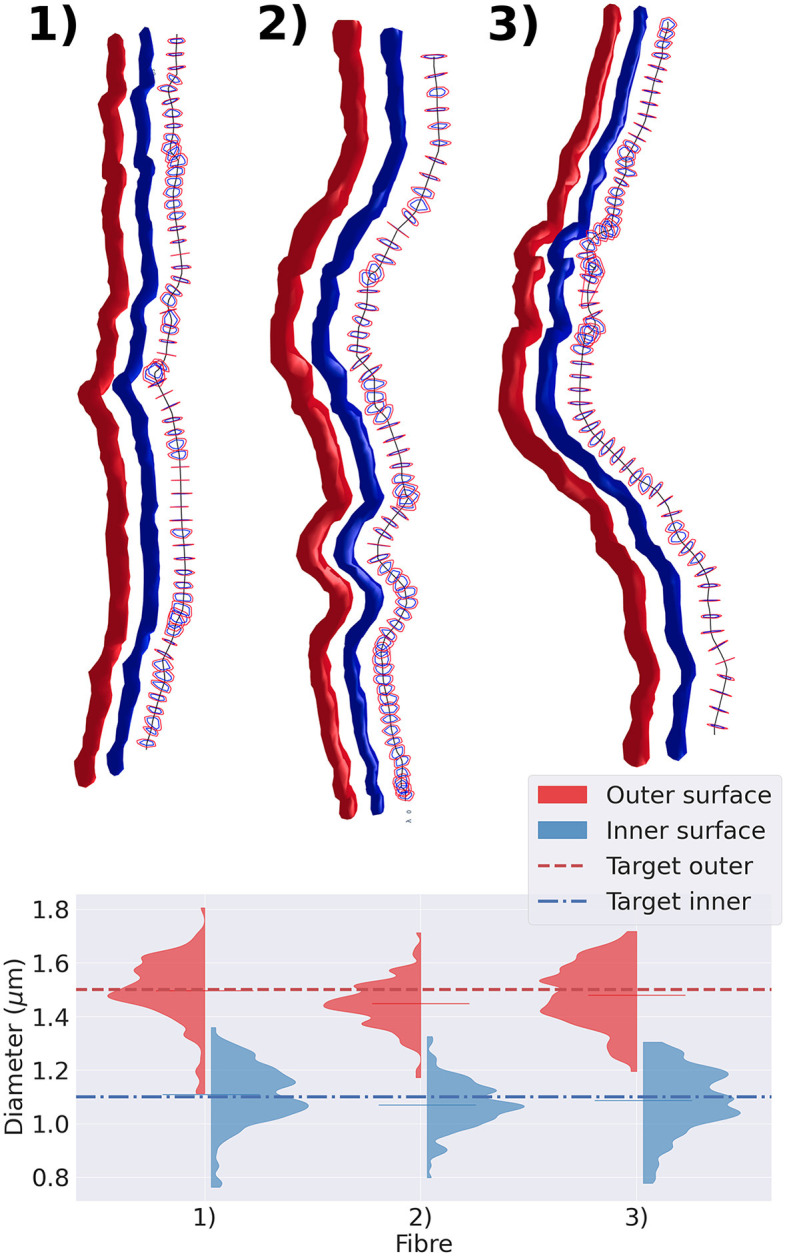
Representative group of three fibres extracted from the substrate shown in Figure 5. In the top panel, we display the fibres with varying diameters and tortuous trajectories. The straightest fibre is presented on the left, while the most tortuous one is displayed on the right. We display each fibre's outer surface in red and its inner surface in blue. We also show the fibre's skeleton in black and cross-sections orthogonal to the fibre's skeleton. The diameter is measured every 1μm along its trajectory. The cross-section cut of the outer surface is shown in red, and the cross-section cut of the inner surface is shown in blue. The bottom panel presents the violin plots of the outer and inner diameters measured. The red (blue) dotted line represents the target outer (inner) diameter of the three fibres.

We extracted three representative fibre segments from the substrates shown in Figure 5 and displayed them in Figure 7. The top panel of the figure exhibits the cross-sections of the outer and inner surfaces of the fibre, along with the cross-sections of their diameters. The bottom panel shows the diameter distribution of each axon. We observed that, regardless of the tortuosity of the fibre trajectory, the diameter distribution of both the inner and outer diameters of all three cases was centred around the target diameter.

#### 4.2.3. Monte-Carlo diffusion simulations

**Figure 8:**
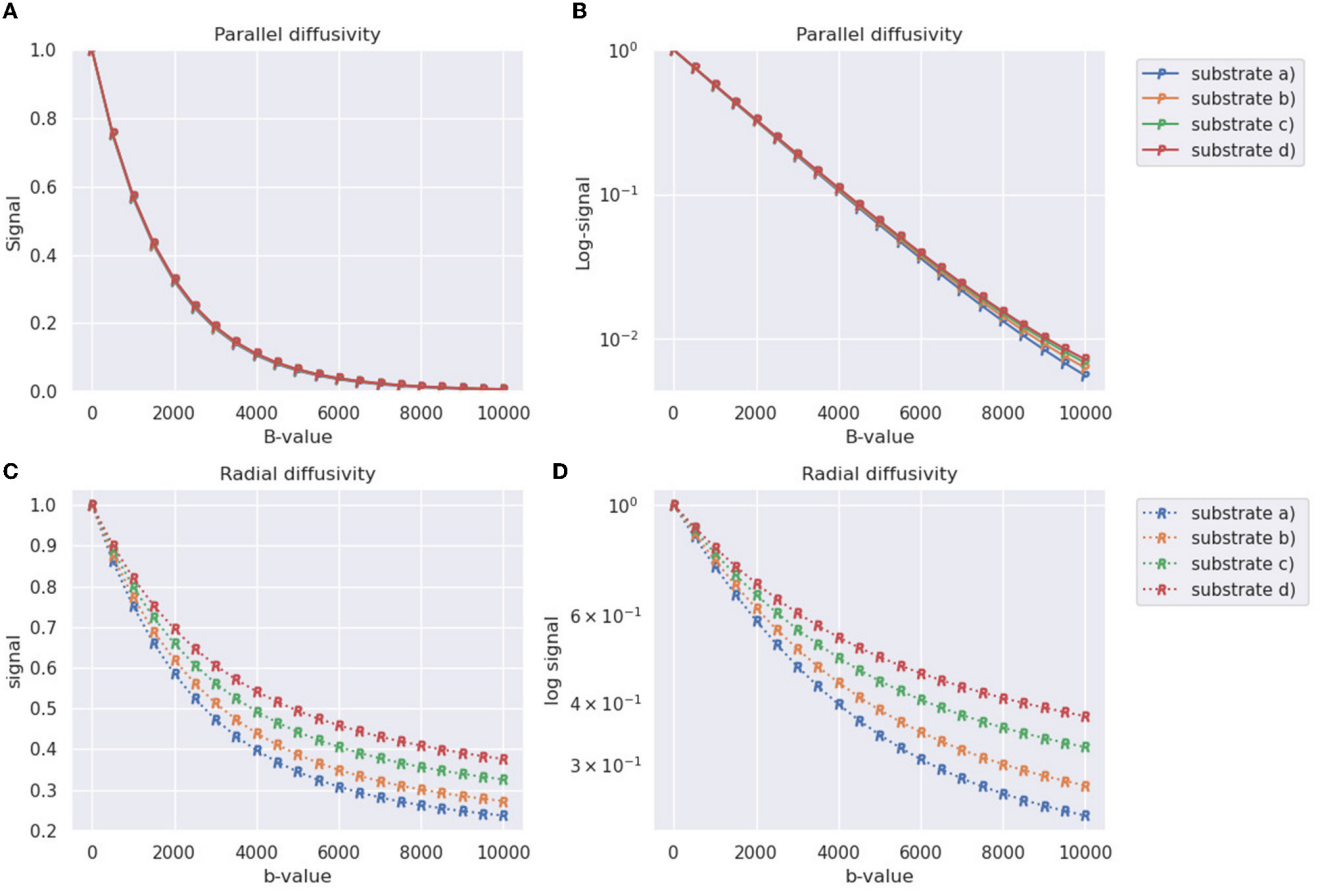
Synthetic DW-MRI signals of substrates generated in Table 2 and shown in Figure 8. The X-axis represents the b-value used for measurement, while the Y-axis represents the normalized signal. **(A)** Parallel signal decay measured along the direction parallel to the substrate fibers (Z-axis). **(B)** Parallel signal decay plot with a logarithmic scale on the Y-axis. **(C)** Radial signal decay averaged over four different diffusion directions orthogonal to the orientation of the fibers. **(D)** Radial signal decay plot with a logarithmic scale on the Y-axis.

The generated signals for the four substrates from Table 2 depicted in Figure 8. In terms of the parallel diffusivity signal decay, Figures 8A, B demonstrate that the four substrates exhibit similar behavior, making it challenging to distinguish them even at *b*-values around 8,000 $\frac{\text{s}}{{\text{mm}}^{2}}$. However, when examining the radial diffusivity signal decay in Figures 8C, D, distinct curves are observed for each substrate. Notably, the logarithmic plot reveals a characteristic tail, indicating the non-Gaussian nature of the diffusion process occurring in the plane perpendicular to the fibre orientations.

## 5. Discussion

Over the last 20 years, Monte-Carlo diffusion simulations have been used to optimise DW-MRI data acquisition protocols and validate microstructure models. Nevertheless, doubts have been raised regarding the accuracy of the simple geometries used to construct the diffusion substrates.

Various tools have been developed to address the challenges associated with substrate complexity, such as MEDUSA (Ginsburger et al., 2019) and CONFIG (Callaghan et al., 2020), each offering distinct approaches to substrate generation. MEDUSA primarily focuses on creating substrates with multiple compartments, including axons, oligodendrocytes, and astrocytes, and also models their interactions within the substrate. The axon compartment encompasses features such as axon diameter distribution, bundle dispersion, local tortuosity, myelin presence, Ranvier nodes, and beading. The oligodendrocytes and astrocytes compartments incorporate parameters such as total diameter distribution, body diameter distribution, number of branchings (processes), and a balancing factor. On the other hand, CONFIG employs biologically motivated rules to model the intricate interactions among axons during growth. Parameters within CONFIG include axon mean radius, standard deviation of radius, bundle dispersion, and packing density. Additionally, it encompasses parameters related to axon growth, such as chemoattraction, fiber collapse, cell-adhesion, and fasciculation.

In this work, we introduced CACTUS, a novel framework to produce numerical substrates mimicking white matter tissue with high volume packings, rich microstructural features and geometries that closely matching the desired input parameters. Among the controllable parameters in CACTUS we include the target distribution for the fibre radii, radii variation per fibre, a myelin compartment, target g-ratio, bundle dispersion, bundle crossings, fibre tortuosity, and packing density. The high versatility of CACTUS is founded on its efficient computational implementation and its mathematical formulation divided into three algorithmic steps (substrate initialization, joint fibre optimization, and fibre radial growth) composed of various competing terms controlling different substrate parameters.

To generate the substrates with CACTUS, we introduced a new algorithm to initialise fibre bundles with a target mean degree of orientation dispersion. Moreover, we introduced a novel capsule-based parametrization for optimizing fibre structures. Compared to circle parametrizations (Close et al., 2009; Ginsburger et al., 2019), the capsule parameterization requires fewer parameters, reducing the complexity of the optimization problem. We adapted the cost functions inspired by Close et al. (2009) and Ginsburger et al. (2019) for capsules and provided analytical derivatives, making the optimization faster and computationally more efficient. Finally, we proposed the fibre radial growth algorithm, which increases the fibre packing density in white matter substrates.

CACTUS was able to enhance the complexity of the fibre microstructure. In particular, our results showed CACTUS can produce substrate with fibre volume fraction beyond the 75% previously achieved. CACTUS reached high fibre volume fractions, up to 95% in its substrates (Table 1). Moreover, it consistently reached fibre volume fractions superior to 90% at all the various levels of bundle dispersion and crossing angles (Table 1, Figure 4).

In the single bundle case, the fibre volume fraction was the highest at 94.7% when fibres were aligned and decreased to 90.8% with increasing mean angular dispersion. Conversely, the fibre volume fraction remained consistently around ~93% in the two-bundle cases, regardless of the crossing angle. However, we note that the packing complexity of substrates with a single bundle and two bundles crossing differs. The former mimics the spatial arrangement of thousands of fibres with different crossing angles, which may produce more empty pockets between fibres and less densely packed substrates.

Another important feature of CACTUS is that it can create substrates with statistical characteristics informed by histological data. Indeed, we can closely adhere to the target statistics of axon volume fraction, myelin volume fraction, and g-ratio reported in histological studies (Stikov et al., 2015; see Figure 5). In all cases, the difference between the target and obtained substrate properties was lower than 2% (see Table 1). Notably, CACTUS is the first tool incorporating the g-ratio as a target characteristic and successfully matching it for large-scale substrates.

Also, CACTUS has the capability to generate substrates with a targeted radii distribution. In our experiments, the approximation of the target distribution improves as substrate size increases, as illustrated in Figure 6, underscoring the importance of generating large substrates. Furthermore, we have the availability to measure fibre geometry accurately. For instance, as seen in Figure 7, the generated fibres have a non-constant longitudinal radius and non-circular cross-sections. Despite the tortuous trajectories of the fibres, the diameter distribution remains centred around the target mean outer (inner) diameter of 1.5μm (1.1μm). Additionally, the diameter distribution presented replicates the diameter variations observed in 3D synchrotron images (Andersson et al., 2020), including longitudinal changes and a lack of skewness.

Finally, while previous works were able to achieve substrate sizes between 30 and 100μm^3^, CACTUS demonstrated a substantial improvement in the generation of larger substrates (Ginsburger et al., 2019; Callaghan et al., 2020). As shown in Figure 6, CACTUS generated substrate sizes ranging from 50 to 500μm^3^, all with up to a 95% fibre volume fraction. Our tool's ability to generate larger substrate sizes is advantageous for Monte-Carlo diffusion simulations in DW-MRI as it has been shown in previous studies (Rafael-Patino et al., 2020), that substrate sizes larger than 200μm^3^ can reduce the sampling bias caused by smaller substrate sizes, potentially leading to more accurate DW-MRI numerical simulations (Romascano et al., 2018; Rafael-Patino et al., 2020). In addition, the ability to generate large substrate sizes is advantageous as DW-MRI modelling is moving toward incorporating more microstructure features such as somas, astroglia, and vascularity (Dyer et al., 2017; Lin et al., 2018; Schneider-Mizell et al., 2021). This makes the generation of large substrates essential for capturing these additional features and moving toward more accurate and comprehensive microstructure imaging.

### 5.1. Limitations and future work

Although CACTUS incorporates complex microstructural features required to mimic some of the most relevant white matter geometrical properties, it still requires fibre-modelling assumptions to reduce the computational burden. Also, CACTUS generates substrates with characteristics resembling those from healthy white matter, but generating pathological tissue requires additional work, which we reserve for future studies.

Additionally, CACTUS focuses solely on generating white matter fibre structures. However, its capacity to generate large substrate sizes expands the potential for including other tissue components in future studies, such as astrocytes, oligodendrocytes, microglia, and capillaries.

Finally, although CACTUS output substrates are suitable for simulators like the MCDC (Rafael-Patino et al., 2020), a thorough analysis is necessary to comprehend the influence of mesh quality, like the number of triangles, on the DW-MRI signals generated by Monte-Carlo simulation. Such analysis is crucial for developing computationally viable simulations.

### 5.2. Applications beyond diffusion MR

The applications of CACTUS are not limited to studying white matter microstructure using DW-MRI. For instance, it can be applied in DW-MRI studies outside the brain (Adelnia et al., 2019), where muscle fibres are organized into fascicles. The microscopic arrangement of muscle fibres can vary between different muscle groups, regions of the same muscle, and multiple pathological conditions (Berry et al., 2018). Moreover, the fibre meshes generated by CACTUS could be used in other applications, like Polarized Light Imaging (PLI; Menzel et al., 2015; Amunts and Axer, 2019, a technique used to infer the local fibre orientation in histological brain sections based on the birefringent properties of the myelin sheaths. The limitations of the birefringence PLI model were investigated in Menzel et al. (2015) by generating synthetic PLI data from a hexagonal bundle of straight parallel cylindrical fibres. Although a more general fibre constructor was recently proposed for validating 3D-PLI techniques (Amunts and Axer, 2019), the white matter substrates generated in our study could provide more realistic geometries for conducting similar studies.

## 6. Conclusion

The generation of realistic substrates is critical for validating DW-MRI models, as it allows researchers to simulate and analyse the effect of microstructural changes on the DW-MRI signal.

In this work, we introduced CACTUS, a novel framework for generating axonal-like substrates with predefined geometrical features of interest. Our experiments show that CACTUS can generate white matter substrates with the desired spatial dimensions, fibre radii, g-ratio, non-circular cross-sections, tortuous trajectories, smooth surfaces, predefined inter-fibre angles and fibre dispersion. Notably, the generated fibre substrates reached up to 95% fibre volume fraction, the highest density reported in the literature to date, in agreement with previous histology studies. We also generated the large substrates/voxels of up to 500μm^3^, with dimensions similar to or higher than those used in preclinical MRI scanners, reducing the gap between numerical and real voxel sizes.

In conclusion, the CACTUS substrate generator tool presented in this study has the potential to advance white matter microstructure modelling. It provides a versatile and customisable platform for generating fibre substrates with quantifiable geometrical characteristics. It is open-source and accessible to the broader research community at: http://cactus.epfl.ch, facilitating the validation and comparison of current and future DW-MRI models.

## Data availability statement

The datasets presented in this study can be found in online repositories. The names of the repository/repositories and accession number(s) can be found at: http://cactus.epfl.ch.

## Author contributions

JV-H contributed to the methodology, coding, simulations, analysis, writing, and visualization. RG contributed to the discussion about substrate generation, the choice of simulation parameters, and writing and reviewing. EC-R and GG contributed to the discussion, methodology, experimental design, and writing review and editing. EF-G contributed to the discussion, methodology, and writing review and editing. J-PT supervised the project, provided funding, contributed to the writing review, and participated in discussions. JR-P contributed to the methodology, experimental design, discussions about simulations, actively contributed to the analysis of results, provided supervision, and writing review and editing. All authors contributed to the article and approved the submitted version.
