# Supplementary material for: CACTUS: a computational framework for generating realistic white matter microstructure substrates
Source: Front Neuroinform. 2023 Aug 1;17:1208073. doi: 10.3389/fninf.2023.1208073 (PMC10434236; doi:10.3389/fninf.2023.1208073)
Supplement: Supplementary file 1 [file Data_Sheet_1.PDF]

CACTUS Supplementary material

1 Joint fibre optimisation

1.1 Derivative of the Overlapping cost function

We define te cost function as follows:

$$f_1(\mathbf{p_0}, \mathbf{p_1}, \mathbf{q_0}, \mathbf{q_1}, t_p, t_q, r_p, r_q) = U^2VWR \tag{1}$$

where

$$\begin{aligned}
\mathbf{Z} &:= (1-t_p)\mathbf{p_0} + t_p\mathbf{p_1} - (1-t_q)\mathbf{q_0} - t_q\mathbf{q_1} && (2)\\
U &= \left(\frac{\|\mathbf{Z}\|}{r_p + r_q} - 1\right) && (3)\\
V &= \|\mathbf{p_1} - \mathbf{p_0}\| && (4)\\
W &= \|\mathbf{q_1} - \mathbf{q_0}\| && (5)\\
R &= r_p r_q && (6)\\
& && (7)
\end{aligned}$$

and the partial derivatives are:

$$\begin{aligned}
\nabla_{p_0} f_1 &= (\frac{\partial U^2}{\partial p_0} V + U^2 \frac{\partial V}{\partial p_0})WR && (8)\\
\nabla_{p_1} f_1 &= (\frac{\partial U^2}{\partial p_1} V + U^2 \frac{\partial V}{\partial p_1})WR && (9)\\
\nabla_{p_1} f_1 &= (\frac{\partial U^2}{\partial r_p} VWR + U^2 VW \frac{\partial R}{\partial r_p} && (10)
\end{aligned}$$

where

$$\frac{\partial U^2}{\partial \mathbf{p_0}} = 2U \frac{1}{r_p + r_q} \frac{1}{\|\mathbf{Z}\|}(1 - t_p)\mathbf{Z} \tag{11}$$

$$\frac{\partial U^2}{\partial \mathbf{p_1}} = 2U \frac{1}{r_p + r_q} \frac{1}{\|\mathbf{Z}\|} t_p \mathbf{Z} \tag{12}$$

$$\frac{\partial V}{\partial \mathbf{p_0}} = \frac{\mathbf{p_0} - \mathbf{p_1}}{\|\mathbf{p_1} - \mathbf{p_0}\|} \tag{13}$$

$$\frac{\partial V}{\partial \mathbf{p_1}} = \frac{\mathbf{p_1} - \mathbf{p_0}}{\|\mathbf{p_1} - \mathbf{p_0}\|} \tag{14}$$

$$\frac{\partial U^2}{\partial r_p} = 2U \frac{-\|\mathbf{Z}\|}{(r_p + r_q)^2} \tag{15}$$

$$\tag{16}$$

1.2 Other Cost functions definitions and derivatives

- $F_{overlap}$: penalises intersection between fibre.
- F_{lenght}: penalises fibre elongations.
- F_{smooth_rad}: Enforces subsequent smooth changes in capsule radii from the same fibre.
- $F_{curvature}$: Penalizes sharp curvature changes between capsules.

$$F_{overlap}(S^{(1)}, \ldots, S^{(k)}) = \sum_{a}^{M} \sum_{b \neq a}^{M} \left[\sum_{i}^{m} \sum_{j}^{m} \boldsymbol{f_1} \left(\mathbf{x_i^a}, \mathbf{x_{i+1}^a}, \mathbf{x_j^b}, \mathbf{x_{j+1}^b}, r_i^a, r_{i+1}^a, r_j^b, r_{j+1}^b; t_{a_i}, t_{b_j} \right) \right] \tag{17}$$

Regularizers: cost functions as constraints In the minimization problem, the overlapping cost function arbitrarily removes collisions. In order to maintain a consistent substrate structure, we introduce other cost functions that act as regularizers.

The length cost function constitutes the primary regularise. The aim is to maintain a capsule length as close as possible to the original.

Since the size of the capsules changes with each iteration of the minimisation procedure, the size of some capsules will change more easily than others (depending on the local environment and the number of capsule collisions). Then, this cost gives a capsule the properties of a spring, such that if its size changes excessively, its neighbours will also change their size to obtain a consistent capsule size throughout the fibre.

This cost function is defined as: **Length cost function** The length cost function definition for a single capsule in an $\mathcal{S}^a$ is inspired by the spring energy from Hooke's law:

$$f_{length}\left(\mathbf{x_i^a}, \mathbf{x_{i+1}^a}, r_i^a, r_{i+1}^a\right) = \frac{2}{r_i^a + r_{i+1}^a} \left(\|\mathbf{x_i^a} - \mathbf{x_{i+1}^a}\| - L_a \right)^2 \tag{18}$$

and L_a is a **constant** defined by the initial length of the fibres. $L_a = \frac{\|\mathbf{x_0^a} - \mathbf{x_{m-1}^a}\|}{m}$.

Then the global length cost function is the sum cost function of all capsules in a fibre.

$$F_{length}(\mathcal{S}^1, \ldots, \mathcal{S}^n) = \sum_{a=1}^{n} \left[\sum_{i=1}^{m^a - 1} f_{length}\left(\mathbf{x_i^a}, \mathbf{x_{i+1}^a}, r_i^a, r_{i+1}^a\right) \right] \tag{19}$$

Derivatives Length Function For simplicity we defined $x := x_i^a$, and $y := x_{i+1}^a$

$$\frac{\partial f_{length}}{\partial \mathbf{x_i^a}} = \frac{4}{r_i^a + r_{i+1}^a} \left(\|\mathbf{x_i^a} - \mathbf{x_{i+1}^a}\| - L\right) \frac{\mathbf{x_i^a} - \mathbf{x_{i+1}^a}}{\|\mathbf{x_i^a} - \mathbf{x_{i+1}^a}\|} \tag{20}$$

$$\frac{\partial f_{length}}{\partial r_i^a} = \frac{-2}{\left(r_i^a + r_{i+1}^a\right)^2} \left(\|\mathbf{x_i^a} - \mathbf{x_{i+1}^a}\| - L\right)^2 \tag{21}$$

1.2.1 Curvature function

Like the length cost function, the curvature cost function is a regulariser that penalises sharp angles in subsequent capsules. Thus, by applying this cost function, we enforce smooth changes across the capsule chain.

Curvature cost function The curvature cost associated between two continuous capsules from the same fibre $S^{(n)}$ is defined as:

$$f_{curve}\left(\mathbf{x_i^a}, \mathbf{x_{i+1}^a}, \mathbf{x_{i+2}^a}, r_i^a, r_{i+1}^a, r_{i+2}^a\right) = \left(1 - \frac{\mathbf{v_i^a} \cdot \mathbf{v_{i+1}^a}}{\|\mathbf{v_i^a}\|\|\mathbf{v_{i+1}^a}\|}\right)^2 \frac{1}{r_i^a + r_{i+1}^a + r_{i+2}^a} \tag{22}$$

where

$$\mathbf{v_i^a} = \mathbf{x_{i+1}^a} - \mathbf{x_i^a} \quad i = 1, 2, 3 \ldots, m \tag{23}$$

are the orientation vectors of the capsules.

Then, measure the global curvature cost function, which is the sum of the cost function between adjacent capsules in each fibre for all fibres.

$$F_{curve}(\mathcal{S}^1, \ldots, \mathcal{S}^n) = \sum_a^n \left[\sum_{i=1}^{m_a - 2} \boldsymbol{f_2}\left(\mathbf{x_i^a}, \mathbf{x_{i+1}^a}, \mathbf{x_{i+2}^a}, r_i^a, r_{i+1}^a, r_{i+2}^a\right)\right] \tag{24}$$

Gradient Elemental cost function: Curvature

$$f_{curve}(\mathbf{x}, \mathbf{y}, \mathbf{z}, r1, r2, 3) = \left(1 - \frac{(\mathbf{x} - \mathbf{y}) \cdot (\mathbf{y} - \mathbf{z})}{\|\mathbf{y} - \mathbf{x}\|\|\mathbf{z} - \mathbf{y}\|}\right)^2 \frac{1}{r_1 + r_2 + r_3} \tag{25}$$

using the following properties:

$$\frac{\partial}{\partial \mathbf{x}}(\mathbf{a} \cdot \mathbf{x}) = \mathbf{a}, \quad \frac{\partial}{\partial \mathbf{x}}\left(\|\mathbf{x}\|_2\right) = \frac{\mathbf{x}}{\|\mathbf{x}\|_2}, \quad \frac{\partial}{\partial \mathbf{x}}\left(\|\mathbf{x}\|_2^{-1}\right) = -\frac{\mathbf{x}}{\|\mathbf{x}\|_2^3}$$

and defining the following variables:

$$\begin{aligned} |\vec{\mathbf{yx}}| &:= \|\mathbf{y} - \mathbf{x}\|, \\ A &:= (\mathbf{y} - \mathbf{x}) \cdot (\mathbf{z} - \mathbf{y}), \\ B &:= \left(\frac{A}{|\vec{\mathbf{yx}}|\,|\vec{\mathbf{zy}}|} - 1\right) \frac{1}{r_1 + r_2 + r_3}, \end{aligned}$$

we can compute the results for the partial derivatives with respect to $\mathbf{x}, \mathbf{y}, \mathbf{z}, r_1, r_2, r_3$

$$\frac{\partial}{\partial \mathbf{x}} f_2 = 2B\left(\frac{(\mathbf{y}-\mathbf{z})}{|\vec{\mathbf{yx}}|\,|\vec{\mathbf{zy}}|} + \frac{A}{|\vec{\mathbf{zy}}|}\frac{(\mathbf{y}-\mathbf{x})}{|\vec{\mathbf{yx}}|^3}\right) \tag{26}$$

$$\nabla_y f_2 = \frac{2B}{|\vec{\mathbf{yx}}|\,|\vec{\mathbf{zy}}|}\left((\mathbf{z}+\mathbf{x}-\mathbf{2y}) + \frac{A\,(\mathbf{z}-\mathbf{y})}{|\vec{\mathbf{zy}}|^2} + \frac{A\,(\mathbf{x}-\mathbf{y})}{|\vec{\mathbf{yx}}|^2}\right) \tag{27}$$

$$\nabla_{\mathbf{z}} f_2 = 2B\left(\frac{(\mathbf{y}-\mathbf{x})}{|\vec{\mathbf{yx}}|\,|\vec{\mathbf{zy}}|} + \frac{A}{|\vec{\mathbf{zy}}|}\frac{(\mathbf{y}-\mathbf{z})}{|\vec{\mathbf{zy}}|^3}\right) \tag{28}$$

$$\nabla_{r_i} f_2 = -B^2 \tag{29}$$

1.3 Fixed radii cost function

In the previous cost functions, the position capsule parameters are updated to hold specific values. Now, we introduce a cost function that regularises the radii parameters of the capsules. At each iteration step of the optimisation procedure, when there is overlapping, the capsule will have two options: to move its position until there is no collision or to reduce its volume to remove collisions. In our optimisation, we prioritize moving the capsule rather than reducing its volume, making the capsule volume reduction the last alternative to solve collision.

With this cost function, we monitor the radii sizes of the capsules to maintain values similar to the original radii preassigned to the fibre.

Another way to

$$f_{fix-radii}(S^{(1)},\ldots,S^{(M)}) = \sum_{n}^{M}\left[\sum_{i=1}^{K}\left(r_i^{(n)} - R_i^{(m)}\right)\right] \tag{30}$$

Derivative fixed radii

$$\frac{\partial f_{sr}}{\partial r_i^a} = 2\left(r_i^a - r_{i+1}^a\right) \tag{31}$$

1.3.1 Smooth Radii cost function

The final regulariser included is the smooth radii cost function, which is applied to one single capsule. Its purpose is to have a similar radius at the extremities of the capsule. As a result, similar to the smooth changes in curvature, we enforce smooth changes in radii between capsules.

Smooth radii cost function The smooth radii cost function definition for a single capsule in a fibre $\mathcal{S}^a$ is based on the quadratic difference of the radii at the beginning and end of the capsule:

$$f_{sr}\left(\mathbf{x_i^a}, \mathbf{x_{i+1}^a}, r_i^a, r_{i+1}^a\right) = \left(r_i^a - r_{i+1}^a\right)^2 \tag{32}$$

Then, the global smooth radii cost function is the sum of the cost function for all capsules in a fibre.

$$F_{somth-radii}(\mathcal{S}^1,\ldots,\mathcal{S}^n) = \sum_{a=1}^{n}\left[\sum_{i=1}^{m_a-1} \boldsymbol{f_{sr}}\left(\mathbf{x_i^a}, \mathbf{x_{i+1}^a}, r_i^a, r_{i+1}^a\right)\right] \tag{33}$$
